# Suitability of Secondary PEEK Telescopic Crowns on Zirconia Primary Crowns: The Influence of Fabrication Method and Taper

**DOI:** 10.3390/ma9110908

**Published:** 2016-11-08

**Authors:** Susanne Merk, Christina Wagner, Veronika Stock, Marlis Eichberger, Patrick R. Schmidlin, Malgorzata Roos, Bogna Stawarczyk

**Affiliations:** 1 Department of Prosthodontics, Dental School, Ludwig-Maximilians-University Munich, Goethestrasse 70, Munich 80336, Germany; merk.susanne@googlemail.com (S.M.); chrissy.wagner@gmx.net (C.W.); stock.veronika@web.de (V.S.); marlis.eichberger@med.uni-muenchen.de (M.E.); 2 Clinic of Preventive Dentistry, Periodontology and Cariology, Center of Dental Medicine, University of Zurich, Plattenstrasse 11, Zurich 8032, Switzerland; Patrick.Schmidlin@zzm.uzh.ch; 3 Division of Biostatistics, Epidemiology Biostatistics and Prevention Institute, University of Zurich, Hirschengraben 84, Zurich 8001, Switzerland; mroos@ifspm.uzh.ch

**Keywords:** polyetheretherketone (PEEK), zirconia, telescopic crowns, computer-aided design/computer-aided manufacturing (CAD/CAM), retention load (RL)

## Abstract

This study investigates the retention load (RL) between ZrO_2_ primary crowns and secondary polyetheretherketone (PEEK) crowns made by different fabrication methods with three different tapers. Standardized primary ZrO_2_ crowns were fabricated with three different tapers: 0°, 1°, and 2° (*n* = 10/group). Ten secondary crowns were fabricated (i) milled from breCam BioHPP blanks (PM); (ii) pressed from industrially fabricated PEEK pellets (PP) (BioHPP Pellet); or (iii) pressed from granular PEEK (PG) (BioHPP Granulat). One calibrated operator adjusted all crowns. In total, the RL of 90 secondary crowns were measured in pull-off tests at 50 mm/min, and each specimen was tested 20 times. Two- and one-way ANOVAs followed by a Scheffé’s post-hoc test were used for data analysis (*p* < 0.05). Within crowns with a 0° taper, the PP group showed significantly higher retention load values compared with the other groups. Among the 1° taper, the PM group presented significantly lower retention loads than the PP group. However, the pressing type had no impact on the results. Within the 2° taper, the fabrication method had no influence on the RL. Within the PM group, the 2° taper showed significantly higher retention load compared with the 1° taper. The taper with 0° was in the same range value as the 1° and 2° tapers. No impact of the taper on the retention value was observed between the PP groups. Within the PG groups, the 0° taper presented significantly lower RL than the 1° taper, whereas the 2° taper showed no differences. The fabrication method of the secondary PEEK crowns and taper angles showed no consistent effect within all tested groups.

## 1. Introduction

In prosthetic dentistry, metal and alloys are the most commonly approved materials [1]. Due to their excellent physico-mechanical properties, precious and non-precious metals are applied in fixed prosthodontics as well as in removable partial prosthodontics such as the double crown technique. While precious metals like gold are particularly well-tolerated, studies have shown that the biocompatibility might be problematic, especially in combination with other metals in the oral cavity [2]. The direct contact of different metals in the oral cavity, as well as metallic ions solved in saliva [3], may cause galvanic corrosion. This problem has been extensively investigated in several studies [2,3,4,5,6]. Even titanium, known for its corrosion resistance [3], may cause corrosion when used in so-called polymetallism [7]. This phenomenon was observed in a primate study with titanium implants combined with superstructures from precious alloys. Titanium can also develop cytotoxic effects, as shown in a recent study [4]. In contrast, the same study mentioned no cytotoxicity of zirconia implants [4].

ZrO_2_, a ceramic material used for medical devices [8], displays good esthetic appearance, high mechanical strength, and high biocompatibility and is used in a wide range of indications, such as frameworks, implants, and abutments [9]. In addition, its very good long-term stability and reliability was proven in a 10-year clinical study [10]. These excellent material properties and the transformation behavior are explained by the yttrium oxide stabilization of ZrO_2_ [8]. ZrO_2_ has also been demonstrated as a material for primary crowns in the double crown technique and has featured itself as an alternative to a gold alloy [11]. In the case of primary crowns with a 0° taper, it even appeared to be better than gold alloys when comparing retention loads [11]. Another investigation of double crowns with different conus angles of 0°–6° [12] concluded that ZrO_2_ primary crowns result in more predictable and less excursive retention loads and that the retention load increased as the conus angle decreased. In addition to the taper, the surface roughness also has an impact on retention load [13], and ZrO_2_ with its low surface roughness is therefore well suitable. Moreover, the low surface roughness and low surface energy result in low biofilm accumulation, which not only applies to ZrO_2_. A study found that implant abutments of PEEK showed equal or lower values of biofilm formation than those made of ZrO_2_ and titanium [14].

Polyetheretherketone (PEEK), a modified polyetherarylketone (PEAK), is a thermoplastic high-performance polymer with a melting point of about 343 °C. The examined physical properties [15], abrasion resistance [16], high hardness, and low water absorption and solubility [17] render this material an interesting material for dentistry. In this field, there are three ways of converting the PEEK material: milling from blanks with computer-aided design/computer-aided manufacturing (CAD/CAM) software, pressing from granules, or pressing from pellets with a special vacuum-pressing device. Blanks and pellets are prepressed forms from the raw material PEEK granules.

PEEK as well as ZrO_2_ represent both very biocompatible materials and are used for several applications, e.g., for dental implants [18], provisional abutments [19], and fixed dental prostheses (FDPs) [20]. However, for extending the field of indications, it was necessary to connect PEEK with other resin composite materials. Despite the resistance to surface modification, a suitable bond can be reached via etching and the use of MMA-containing coupling agents [21]. PEEK is also used for implant-supported bars and clamps for removable prostheses [18,19,22,23,24]. Furthermore, recent publications reported that PEEK is a suitable material for double crown systems [25,26,27]. Finally, a gold alloy with its ductility already affects good results in combination with ZrO_2_ [12]. Therefore, a new concept could be the combination of these two biocompatible materials, i.e., ZrO_2_ and PEEK in order to produce metal-free FDPs such as telescopic crowns. As a matter of fact, to the authors’ knowledge, there are no existing published studies with regard to this topic—especially when aiming to assess the retention load between ZrO_2_ primary crowns and secondary PEEK crowns made by different fabrication methods with three different tapers. The null hypotheses of this study were therefore to test that (i) the fabrication method and (ii) the taper have no influence on the retention load.

## 2. Material and Methods

In this study, the retention load of 90 double crowns was investigated. The primary crowns were made from ZrO_2_ (Ceramill ZI 71; AmannGirrbach AG, Koblach, Austria, LOT: 1303002), whereas the secondary crowns were made from PEEK materials: (i) breCam BioHPP blanks (bredent, Senden, Germany, LOT: 394172) for CAD/CAM milling; (ii) BioHPP Pellet (bredent, Senden, Germany, LOT: 393554) for PEEK pellet pressing; (iii) BioHPP Granulat (bredent, Senden, Germany, LOT: 379806) for PEEK granular pressing. According to the manufacturer BioHPP is a ceramic-reinforced, partly crystalline polyetheretherketone (PEEK).

### 2.1. Fabrication of Specimens

First, a prepared plastic model tooth was used as a template for silicone molds (Adisil blau 9:1, Siladent, Goslar, Germany). Based on this master model, 30 wax (Milling- & Universal Wax blue; GEBDI, Engen, Germany) duplicates were manufactured and cast in a base metal alloy (Remanium GM800+; Dentaurum, Ispringen, Germany, LOT: 936) using a conventional casting method. These molar dies were scanned (Ceramill map 300, AmannGirrbach AG), and, based on these data sets, three master type primary crowns with tapers of 0°, 1°, and 2° were constructed (Ceramill mind, AmannGirrbach AG). All three constructions were designed with wall thicknesses of 2 mm. The samples with a taper of 0° received a chamfer preparation. Having milled each of the 10 primary crowns with 0°, 1°, and 2°, (Ceramill Motion 2 System, AmannGirrbach AG), these 30 ZrO_2_ primary crowns were sintered (Ceramill therm, AmannGirrbach AG) with the following program: a heat-up phase to a final temperature of 1450 °C (heating rate 5–10 K/min), 2 h dwell time at this temperature, and finally a cooling phase to room temperature (at least <200 °C), approx. 5 K/min.

The next step was an adhesive cementation process of the ZrO_2_ primary crowns on the metal dies with self-adhesive resin cement (RelyX Unicem 2, 3M ESPE, Seefeld, Germany, LOT: 509981). Afterwards, they were aligned in a parallel fashion and based in a plaster socket to ensure their ideal position during pull-off testing. Afterwards, they were all manually reworked in a surveyor device (parallelometer F4 basic, DeguDent, Hanau, Germany) by a calibrated operator to ensure their individual taper angles. Therefore, diamond burs with appropriate tapers and a turbine (W & H Perfecta 900, W & H Dentalwerk Bürmoos GmbH, Bürmoos, Austria) were used under constant water cooling. The diamond processing occurred gradually from coarse (grit size 151 µm) to middle (grit size 107 µm) to fine grit (grit size 46 µm; Ceramic Art Set 4371/4369, ZR374M/F, Komet Dental, Lemgo, Germany; all grit sizes according to the manufacturer’s data). The surface was first polished with a 3-step silicone polish system (Ceramic Art Set 4371, Komet Dental) and finished with round brushes (Komet Dental, REF: 9638900190) and polishing paste (YETI DIA-GLACE; YETI Dentalprodukte GmbH, Engen, Germany, Pat. 3832085.1) for a high gloss. Due to this individualization, 30 primary crowns resulted, and each one was individually scanned (Arti-Spray, white, BK 285; Dr. Jean Bausch KG, Cologne, Germany; Ceramill map 300, AmannGirrbach AG).

**Figure 1:**
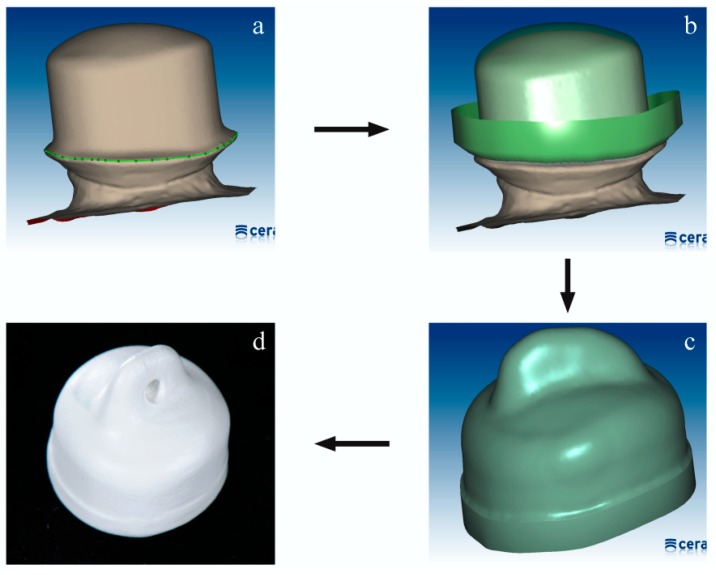
Photo series about CAD/CAM processing a PEEK secondary crown (PM) (**a**) scanned primary crown (0° taper), marked preparation border; (**b**) secondary crown construction; (**c**) setup-wizard of secondary crown with designed retainer; (**d**) milled secondary crown, end result.

On this basis, constructions for the secondary crowns were designed and milled out of these data (ZENO Tec System, ZENO 4030 M1, Wieland + Dental, Pforzheim, Germany), i.e., 30 specimens from PEEK blanks (Figure 1) (breCAM BioHPP, bredent, LOT: 394172) (PM) and 60 specimens from wax blanks (breCAM.wax; bredent, LOT: 382697). The latter were randomly divided in two groups—30 specimens for the production from pellets (PP) and 30 specimens for the production from granules (PG).

**Figure 2:**
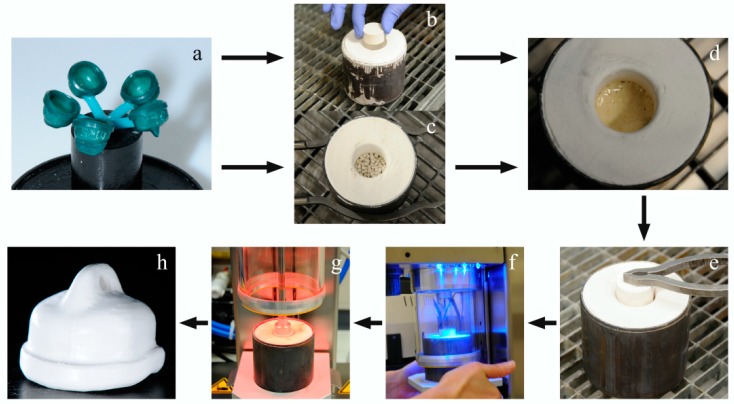
Photo series about processing from wax model to PEEK secondary crown (PP/PG) (**a**) 5 wax models (secondary crowns) on the muffle plate, prepared for embedding; (**b**) one piece PEEK pellet, put into a preheated muffle (melting reservoir); (**c**) PEEK granular filled into a preheated muffle; (**d**) PEEK material in its melted phase (about 380 °C); (**e**) preheated press plunger positioned into the melting reservoir; (**f**) pressing device during manually closing; (**g**) ending of the pressing process: pressure was kept while cooling down; (**h**) pressed PEEK secondary crown, end result.

Afterwards, they were embedded in a muffle (5–6 wax models together) with a mixing ratio of 70% liquid/distilled water (Bresol for 2 press liquid, bredent, LOT: 1; Brevest for 2 press, bredent, LOT: 1) for 25 min according to the manufacturer’s instructions. The muffle and a press plunger (for 2 press filler, bredent, LOT: 397014) were placed in a 850 °C preheated furnace for 60 min, which was cooled down afterwards with 8 °C/min to 400 °C. After a waiting period of 20 min at this temperature, PEEK granular and pellets (Bio HPP Granulat/Pellet, bredent) were filled into the melt reservoir of the muffle. The melting period accounted for 20 min. The muffle with the melted PEEK and the positioned press plunger was placed onto the pressing table of the pressing device and was manually closed (vacuum pressing device for 2 press; bredent). The vacuum press process with a pressure of 4.5 bar ran automatically (Figure 2).

After divesting all specimens, they were air-abraded (Fine-blaster type FG 3, Sandmaster, Zofingen, Switzerland) with 50 µm of Al_2_O_3_ (Hasenfratz, Sandstrahltechnik, Aßling, Germany) at a pressure of 2 bar. Fitting of every secondary crown (PM/PP/PG) to their primary crown was tested by a calibrated operator using articulation spray (Arti-Spray, white, BK 285, Dr. Jean Bausch KG, Cologne, Germany) and adjusted using cross-cut burs (Komet Dental, LOT: 277889) where necessary. Silicone polishers (Ceragum Wheel, bredent, REF: PRKM22000) and polishing brushes (Komet Dental, LOT: 226983) with polishing paste (Abraso-Starglanz asg, bredent REF: 52000163) were used to finish the specimens.

### 2.2. Retention Load Measurement

**Figure 3:**
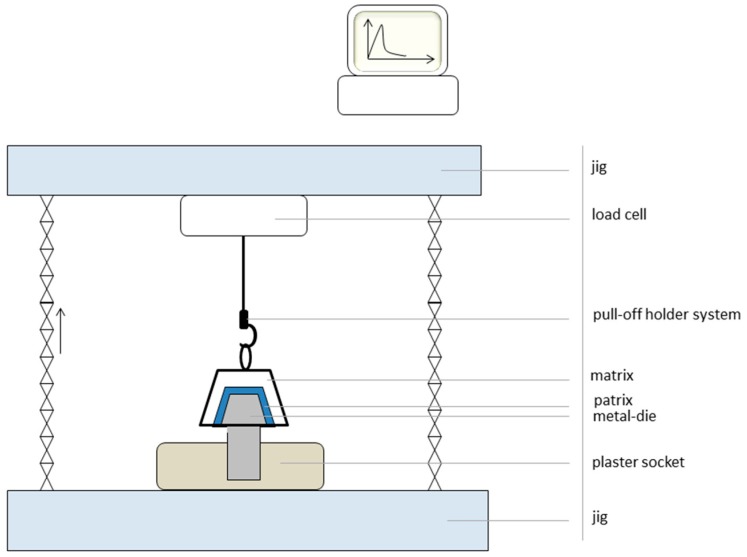
Test design.

For retention load measurement, a pull-off test setup was created (Figure 3). The socketed die with its primary crown was fixed in a universal testing device (Zwick 1445, Zwick, Ulm, Germany). The secondary crown was wetted with artificial saliva (Glandosane, cell pharm GmbH, No. 9235461109) and placed in a final position onto the respective primary crown. In each of the 20 cycles, a 5 kg weight was put on top for 20 s to ensure a comparable starting situation for each specimen. The secondary crown was pulled off with a speed of 50 mm/min [25,26,27,28,29] using a hook that was mounted in the designed retainer.

### 2.3. Statistical Analyses

A Kolmogorov–Smirnov test was used to verify the normality of data distribution. Descriptive statistics (mean, standard deviation (SD), 95% confidence intervals (CI), minimum, median, and maximum) were computed. Significant differences between the groups were tested with 2-way and 1-way ANOVAs, followed by the Scheffé’s post-hoc test. All statistical tests were calculated using IBM SPSS (Version 20; IBM Corporation, Armonk, NY, USA) (*p* < 0.05).

## 3. Results

The cycles presented no impact on the resulting measurements of each specimen (*p* = 0.354); therefore, arithmetic means were computed. The descriptive statistics are summarized in Table 1. The Kolmogorov–Smirnov test indicated no evidence of a violation of the normality assumption in the data (*p* < 0.05). According to the two-way ANOVA, the results showed that the fabrication methods (*p* = 0.144) as well as the taper type (*p* = 0.958) had no effect on the retention load results. However, the interaction between both parameters was significant (*p* = 0.001). Subsequently, the data was split and analyzed with respect to the test hypotheses individually (Table 1).

By comparison of the fabrication method within 0° crowns showed that the pellet pressed group displayed significantly higher retention load values compared with the other groups (*p* = 0.005). Among the 1° taper, the milled secondary crowns had significantly lower retention load values than the pressed groups (*p* < 0.001). However, the pressing type had no impact on the results. The groups with the 2° taper presented no effect on the fabrication method (*p* = 0.228).

Within milled PEEK secondary crowns, a 2° taper showed significantly higher retention loads compared with a 1° taper (*p* = 0.020). A taper with 0° was in the same range of values as compared to the 1° and 2° taper. No impact of taper on retention values was observed between the pressed secondary crowns from the PEEK pellet material (*p* = 0.658). In contrast, the pressed crowns from the granular 0° taper presented significantly lower values than the 1° ones (*p* = 0.009), whereas the 2° taper showed no differences.

## 4. Discussion

To the authors’ knowledge, no studies of the combination of ZrO_2_ and PEEK with the double crown technique have been published. Therefore, this study was aimed at investigating the retention load of ZrO_2_ primary and PEEK secondary crowns.

The null hypothesis regarding the fabrication method of PEEK material must be rejected since the statistical evaluation showed differences between PM, PP, and PG. For instance, in the 1° taper group, milled PEEK specimens showed significantly lower retention loads than both pressed groups. This might be explained by differing fabrication procedures. On the one hand, there are specimens (primary and secondary crown) influenced only by CAD/CAM fabrication processes (PM); on the other hand, there are specimens (PP/PG) influenced by the pressing process. With CAD/CAM processing, the specimens are less subjected to unpredictable manufacturing aspects and bias. The main fact is to determine software parameters for designing the secondary crowns. In contrast, the pressing process entails passing several steps, such as embedding the wax models, heating the muffle and the PEEK material, letting it cool down, and allowing air-abrasion during the divesting process.

A recent report of a study on the fracture load of FDPs noted an influence of the fabrication method on PEEK material properties [20]. As a result, PEEK granular showed an incomplete fracture and a plastic deformation, in contrast to PEEK blanks and pellets. This study attributed this to the industrial prepressing processes, which increase mechanical properties. PEEK granular passes no prepressing, whereas PEEK blanks and pellets are extruded out of PEEK granular [20]. However, in this investigation, the specimens pressed from PEEK pellets showed a significantly higher retention load compared with those pressed from granules and the milled ones among the 0° taper group. PEEK blanks/granular were heated once. The blanks were heated during industrial fabrication by extruding out of granular and the PEEK granular just before pressing. It is supposed that the two heating processes of PEEK pellets—extruding and heating just before pressing—change the material properties and therefore the telescopic fitting. Due to these facts, it is suggested that the number of heating processes influences the retention load, whereas industrial prepressing considerably influences the fracture load.

The null hypothesis regarding the taper can be rejected because the taper has an impact on the retention load, especially in consideration of the milled and granular groups. Former studies showed that retention load decreases with the increase in the taper [12,30]. In contrast, this pattern could not be observed in this study. A reason could be the low flexural modulus of PEEK material amounting to only 4 GP [15]. This could lead to a growing wedging of primary and secondary crowns in tapered groups, while adapting them with a 5 kg weight, whereas almost no wedging occurs in 0° double crowns due to the parallelism and their chamfer with a final stop. These facts are reflected in lower retention load values of the 0° taper compared with the 1° taper in the granular group on the one hand, and lower retention load values of the 1° taper compared with the 2° taper in the milled group on the other.

Furthermore, in this study the interval between cone angles is altogether only 2 degrees (just 1° each). That is why retention load values regarding the taper are not significantly different in every case. However, a major study concluded that the cone angle of a telescopic crown should be less than 2 degrees for long-term use [30]. It can be suggested that the recommended clinical retention load values may be achieved by reducing the tread area. Nevertheless, the results of the present investigation seem to be suitable for using telescopic crowns, although long-term studies with clinical conditions are required.

## 5. Conclusions

In assessing retention load, PEEK may be a suitable material for removable prosthesis and a telescopic crown technique when used on zirconia crowns. However, long-term investigations and the advancement of PEEK CAD/CAM processing are still necessary.

## Figures and Tables

**Table 1:** Descriptive statistics such as mean with standard deviation (SD), 95% confidence intervals (95% CI), and the robust statistics (minimum/median/maximum). All values for retention load are presented in Newton (N).

Taper Angle	Material Group	Mean ± SD	95% CI	Min/Median/Max
0°	PM	13.83 ± 7.82 ^ab/A^	(8.1; 19.5)	2.8/13.0/28.0
	PP	22.83 ± 5.94 ^a/B^	(18.4; 27.1)	16.9/21.4/33.1
	PG	15.87 ± 2.58 ^a/A^	(13.9; 17.8)	12.5/15.8/20.2
1°	PM	6.07 ± 3.01 ^a/A^	(3.8; 8.3)	1.7/6.8/9.6
	PP	21.06 ± 8.60 ^a/B^	(14.8; 27.3)	11.2/21.9/31.7
	PG	27.00 ± 10.05 ^b/B^	(19.7; 34.2)	11.3/26.9/46.5
2°	PM	14.10 ± 8.19 ^b/A^	(8.1; 20.0)	7.2/11.2/34.7
	PP	19.84 ± 7.13 ^a/A^	(14.6; 25.0)	9.6/18.8/29.4
	PG	19.05 ± 8.25 ^ab/A^	(13.1; 25.0)	5.3/18.4/31.9

PM: PEEK milled; PP: PEEK pressed pellet; PG: PEEK pressed granular; ^a,b^: differences between the taper angles within one material group; ^A,B^: differences between the material group within one particular taper.

## Abbreviations

The following abbreviations are used in this manuscript:

PEEK	polyetheretherketone
PEAK	polyetherarylketone
CAD/CAM	computer-aided design/computer-aided manufacturing
RL	retention load
ZrO_2_	zirconia
MMA	methyl methacrylate
FDPs	fixed dental prostheses
Al_2_O_3_	aluminum oxide
